# Circulating immune cell dynamics in patients with triple negative breast cancer treated with neoadjuvant chemotherapy

**DOI:** 10.1002/cam4.3358

**Published:** 2020-08-05

**Authors:** Sarah Talamantes, Eric Xie, Ricardo L. B. Costa, Melissa Chen, Alfred Rademaker, Cesar A. Santa‐Maria

**Affiliations:** ^1^ Northwestern University Feinberg School of Medicine Chicago IL USA; ^2^ Johns Hopkins University School of Medicine Baltimore MD USA; ^3^ H. Lee Moffitt Cancer Center Tampa FL USA

**Keywords:** circulating immune biomarkers, lymphocytes, monocytes, neoadjuvant chemotherapy, triple negative breast cancer

## Abstract

**Background:**

Lymphopenia has been associated with inferior cancer outcomes, but there is limited data in breast cancer. We describe the effects of neoadjuvant chemotherapy on circulating immune cells and its association with pathological complete response (pCR) rates in triple negative breast cancer (TNBC).

**Methods:**

We constructed a database of patients with early stage TNBC treated with neoadjuvant chemotherapy. Circulating lymphocytes and monocytes were assessed before and after neoadjuvant chemotherapy. These were correlated with pCR rates and disease‐free survival (DFS) using Fisher's exact test, logistic regression, and the log‐rank test.

**Results:**

From 2000 to 2015, we identified 95 eligible patients. Median age was 50; 29 (31%) were treated with platinum‐containing chemotherapy; and 66 (69%) with nonplatinum‐containing chemotherapy (anthracycline‐taxane, or either alone). About 32 (34%) patients achieved a pCR; and 33 (35%) had recurrence events. Median follow‐up time was 47 months. No significant associations were found between changes in lymphocytes and pCR or DFS. There was a correlation between lower monocyte levels after neoadjuvant chemotherapy and pCR (mean monocyte 0.56 in those with no‐pCR vs 0.46 in those with pCR, *P* = .049, multivariate *P* = .078) and DFS (median DFS in highest monocyte quartile was 30 vs 107 months in lowest quartile, *P* = .022, multivariate *P* = .023). In patients who received nonplatinum regimens, DFS was better among those who had larger decreases in monocytes.

**Conclusions:**

Development of lymphopenia from neoadjuvant chemotherapy was not associated with pCR in patients with TNBC. However, lower absolute circulating monocytes after neoadjuvant chemotherapy was associated with improved outcomes.

## Graphical abstract

Development of lymphopenia from neoadjuvant chemotherapy was not associated with pathological complete response in patients with triple negative breast cancer. However, lower absolute circulating monocyte counts after neoadjuvant chemotherapy were associated with improved clinical outcomes.

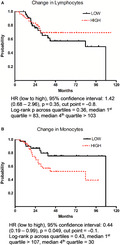

## 1 INTRODUCTION

According to the World Health Organization, there are over 2 million new cases of breast cancer diagnosed each year worldwide, representing the most commonly diagnosed nondermatologic cancer in women.^1^ Of these cases, approximately 15% are triple negative, defined by the absence of estrogen and progesterone receptor (ER and PR, respectively), and human epidermal growth factor receptor 2 (HER2) expression/amplification.^2^ Triple negative breast cancer (TNBC) is typically an aggressive form of breast cancer with a poorer prognosis.^3^, ^4^ Due to lack of targeted therapy options, chemotherapy is the mainstay treatment for TNBC. Chemotherapy can be administered prior to surgery and may result in patients achieving a pathologic complete response (pCR). In patients with TNBC, pCR is positively associated with improved event‐free survival (EFS, HR 0.25; 95% CI, 0.18‐0.34) and overall survival (OS, HR 0.19; 95% CI, 0.12‐0.31).^5^

Chemotherapy requires an intact immune system to exert its full antitumor effect, but can also modify the tumor microenvironment (TME).^6^ Lymphocytes are part of both the adaptive and innate immune system and are responsible for the recognition of foreign antigens, amplification of the immune response and destruction of malignant cells with recognizable neoantigens.^7^, ^8^ Tumor infiltrating lymphocytes (TILs) found in the stroma may approximate an “inflamed” TME; possibly a surrogate for the immunogenicity of the tumor itself. Furthermore, higher TILs correlates with pCR in patients with TNBC and are associated with improved disease‐free survival (DFS), and OS.^9^, ^10^, ^11^, ^12^ Circulating lymphocytes, on the contrary, reflect the host immune system, and lower circulating lymphocyte counts have been linked to both higher recurrence rate in many different types of cancers and poorer response to immune checkpoint blockade.^13^, ^14^

The main purpose of the current study was to evaluate the associations of circulating immune cells dynamics with pCR and DFS in patients with early stage TNBC treated with neoadjuvant chemotherapy.

## 2 METHODS

This is a single‐center, retrospective, cohort study of patients with breast cancer from Northwestern University Robert H. Lurie Comprehensive Cancer Center identified from the Northwestern Medicine Enterprise Data Warehouse. Internal review board (IRB) approval was obtained to retrospectively identify (via our institutional electronic data warehouse) and investigate patient charts. Basic patient data, characteristics, and clinical details were obtained. Further clinical and pathologic information was then collected using the electronic medical record. Patients were sorted and included in the study based on the following eligibility criteria: (A) stage 1‐3 TNBC defined as lack of ER, PR, and HER2 expression/amplification by ASCO/CAP guidelines, (B) treated with neoadjuvant chemotherapy, and (C) had available circulating lymphocyte and monocyte counts at the time of diagnosis, after neoadjuvant chemotherapy, and prior to surgery (all values in K/cu mm).^15^, ^16^

The primary objective of the study was to describe associations between changes in circulating lymphocyte counts before and after neoadjuvant chemotherapy and pCR in patients with early stage TNBC. We assessed interactions between changes in circulating lymphocyte counts with race, ethnicity, age, body mass index (BMI), p53, Ki67, and grade on pCR rate among patients with TNBC treated with neoadjuvant chemotherapy. We assessed correlations between circulating lymphocyte counts and DFS. Similar analyses were also completed with monocyte counts to assess the association between pCR rate and DFS.

We assessed absolute circulating lymphocyte and monocyte counts before and after neoadjuvant chemotherapy, and then, calculated the change in these cell counts by subtracting the number of lymphocytes and monocytes prior to initiation of neoadjuvant chemotherapy from cell counts after completion of chemotherapy (after‐before). Baseline counts and changes in counts were analyzed as continuous variables or as categorical variables split at the median or split into quartiles. The comparison of mean circulating lymphocyte and monocyte counts between pCR positive and negative groups was analyzed by the t test and multiple regression (Figure 2 and Figure S1). The comparison of DFS across categories of circulating monocyte and lymphocyte counts (median split or quartile split) was analyzed by the log‐rank test or proportional hazards regression (Figure 3 and Figure S2). Hazard ratios and 95% confidence intervals were used to quantify relationships. The relationships between circulating monocyte and lymphocyte counts and various clinical and pathologic information were analyzed by Fisher's exact test (median split on age, Ki67 and p53, Table 2). Analyses were done using SAS software (SAS Institute Inc 2012. SAS OnlineDoc^®^ 9.4. Cary, NC: SAS Institute Inc).

## 3 RESULTS

**Figure 1:**
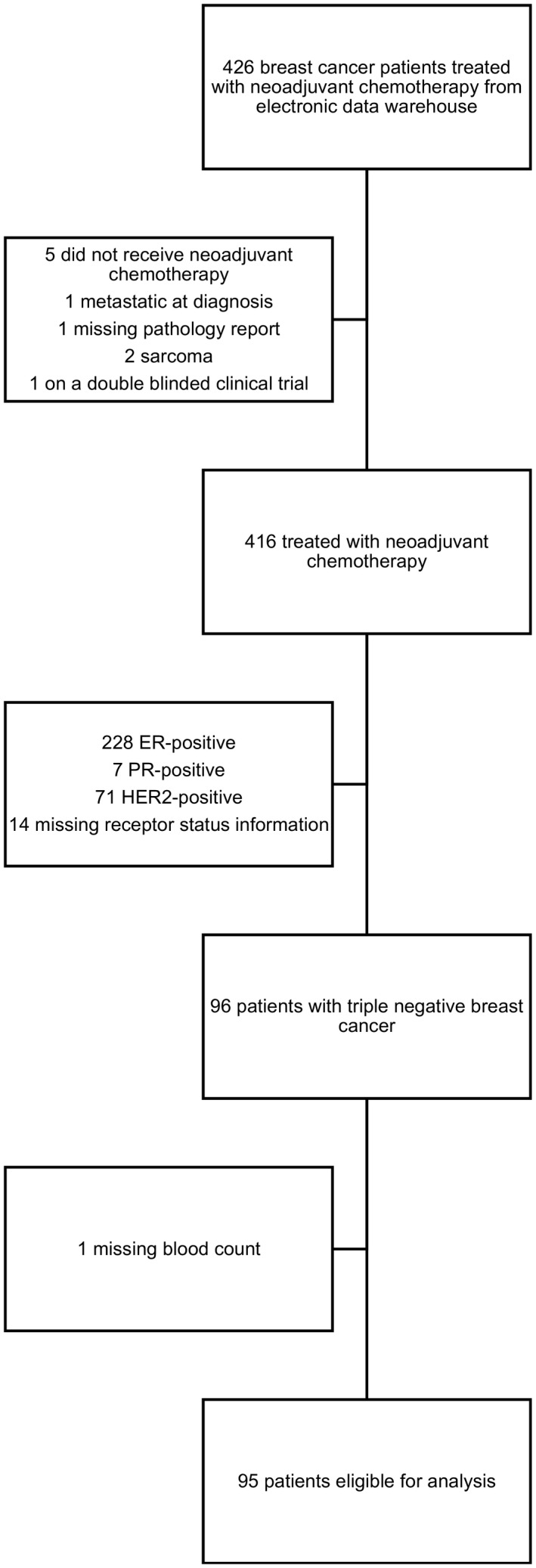
Consort Diagram

From 2000 to 2016, 426 patients with breast cancer were identified by the Northwestern Medicine Enterprise Data Warehouse. Of these, 95 met eligibility criteria and were included in the analysis (Figure 1).

The characteristics of the study population are shown (Table 1). In brief, the median age of patients was 50 years (range 26‐79); 63 (66%) patients were treated with anthracyclines plus taxanes, 29 (31%) were treated with platinum‐based chemotherapy, and 3 (3%) were treated with anthracyclines without taxanes or vice versa; 33 (35%) patients achieved a pCR; and 33 (35%) patients had recurrence events. Median follow‐up time was 47 months (range 13‐123).

**Table 1:** Description of patient population

Characteristic	Number, n = 95 (%)
Age at diagnosis (mean, range)	50 (26‐79)
Race	
Caucasian	45 (47)
African American	28 (29)
Other	15 (15)
Unknown	7 (7)
Ethnicity	
Non‐Hispanic	83 (88)
Hispanic	6 (6)
Unknown	6 (6)
Body Mass Index (BMI)	
Underweight/Normal	26 (27)
Overweight/Obese	69 (73)
Baseline Ki67% (mean, range)	60 (0‐95)
Baseline p53 (mean, range)	44 (0‐100)
Baseline Grade	
1	0 (0)
2	5 (5)
3	86 (95)
Surgery	
Lumpectomy	47 (49)
Mastectomy	48 (51)
Neoadjuvant chemotherapy	
Anthracycline with taxane	63 (66)
Platinum based (± taxane, ± anthracycline)	29 (31)
Anthracycline without taxane	2 (2)
Taxanes only	1 (1)
Pathologic complete response	33 (35)
Recurrences, total	33 (35)
Locoregional	13 (14)
Distant	20 (21)

**Figure 2:**
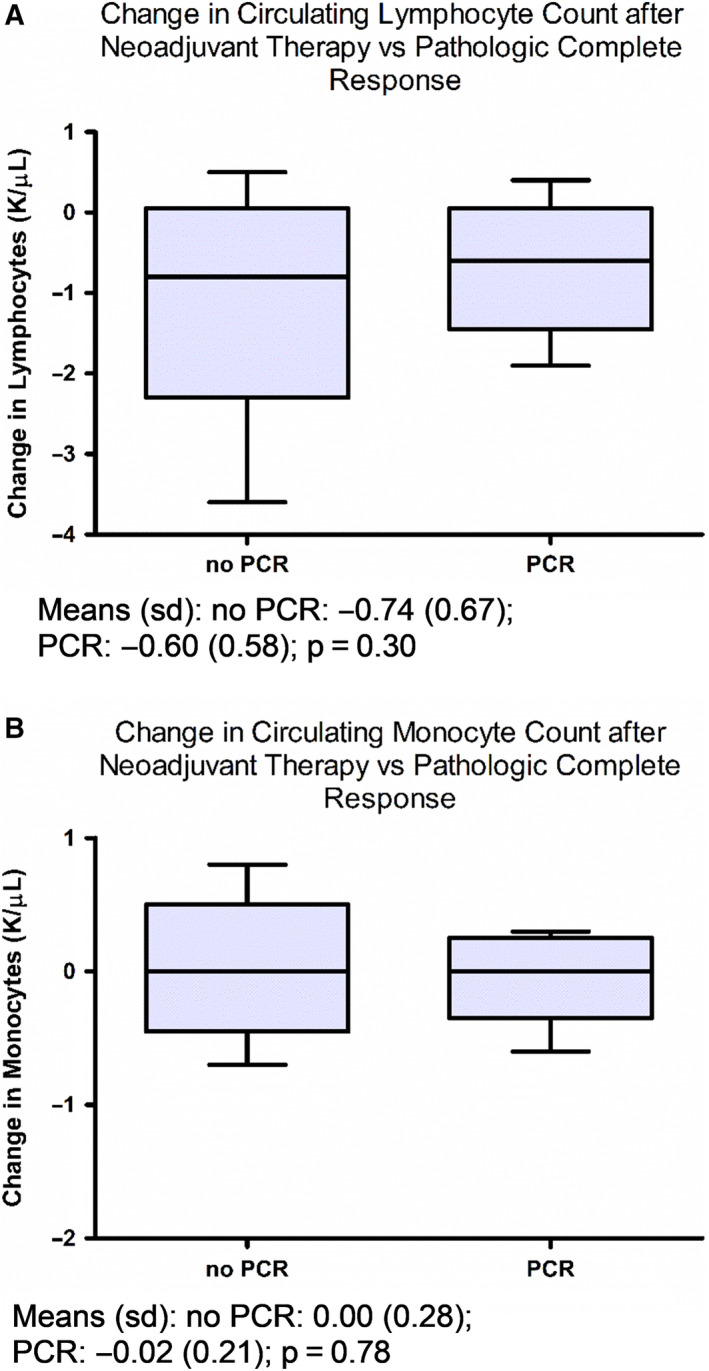
Relationship of changes in circulating lymphocytes or monocytes with pCR. Box plots indicate median, interquartile range, and minimum/maximum. Association of circulating (A) lymphocyte and (B) monocyte count changes before and after neoadjuvant chemotherapy with pCR. Means (SD) and *P*‐values by t test are reported

Figure 2 and Figure S1 gives boxplots of the circulating lymphocyte and monocyte counts and changes for pCR negative and positive groups. No significant associations were found between changes in lymphocyte or monocyte counts and pCR (Figure 2A and B). Mean lymphocyte reduction was 0.74 in those with no‐pCR vs 0.60 in those with pCR, *P* = .30; mean monocyte reduction was 0 in those with no‐pCR vs 0.016 in those with pCR, *P* = .78. There was a significant correlation between monocytes after neoadjuvant chemotherapy and pCR (mean monocyte level was 0.56 in those with no‐pCR vs 0.46 in those with pCR, *P* = .049, Figure S1D). In multivariate analyses adjusting for age and grade, mean monocyte level was 0.61 in those with no‐pCR vs 0.52 in those with pCR, *P* = .078.

**Figure 3:**
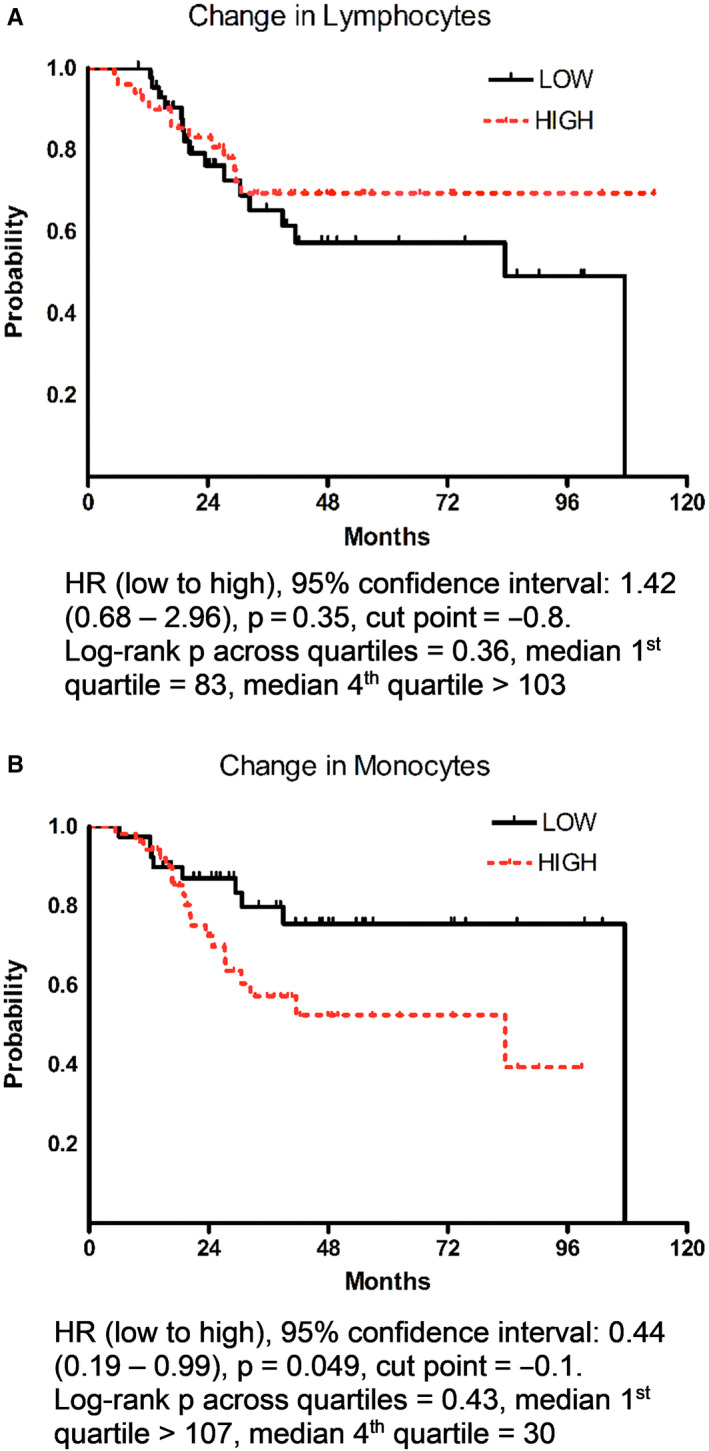
Association of circulating (A) lymphocyte and (B) monocyte count changes before and after neoadjuvant chemotherapy with DFS. Figures show data for LOW (below the median) and HIGH (above the median). Kaplan‐Meier curves, hazard ratios (95% confidence intervals), *P*‐values by proportional hazards regression and median cut point in K/cu mm are reported. Log rank *P*‐values are also reported across all quartiles with median DFS given for the lowest (first) and highest (fourth) quartiles

Figure 3 and Figure S2 gives Kaplan‐Meir curves for DFS when the circulating lymphocyte and monocyte counts and changes were classified as below or above their median. There was no correlation between changes in lymphocytes with DFS (Figure 3A), but changes in monocytes that were below the median (ie, larger decreases after completion of neoadjuvant chemotherapy) resulted in significantly better DFS (Figure 3B). Lower monocytes after neoadjuvant chemotherapy were related to higher DFS (median DFS in highest monocyte quartile was 30 months vs 107 months in the lowest quartile, *P* = .022 across quartiles, Figure S2D). Results remained unchanged when adjusted for age and grade (*P* = .023). Baseline lymphocytes and monocytes did not correlate with pCR or DFS, and lymphocytes after neoadjuvant chemotherapy did not correlate with pCR or DFS.

About 24% of patients treated with platinum‐based regimens and 56% of patients treated with nonplatinum‐based regimens (anthracycline with taxanes or either alone) had lymphocytes decrease below the median (OR 4.01; 95% CI, 1.51‐10.68, *P* = .007) post‐neoadjuvant chemotherapy. About 40% of patients treated with nonplatinum and 50% of patients treated with platinum‐based regimens had monocytes decrease below the median (OR 0.66; 95% CI, 0.27‐1.61, *P* = .37). In addition, 26% of Caucasians had low baseline monocytes vs 50% of African Americans (OR 2.91; 95% CI, 1.06‐7.98, *P* = .045) with low baseline monocyte counts; there were no differences in baseline lymphocyte counts. There was no association between changes in lymphocytes or monocytes with age, BMI, Ki67, or p53 (Table 2).

**Table 2:** Relationship of cohort characteristics with change in circulating lymphocyte and monocyte counts as shown by odds ratio (OR), 95% confidence interval (*P*‐value) using Fisher's exact test

Characteristics	Change in Circulating Lymphocytes (K/uL)	Change in Circulating Monocytes (K/µL)
Age at diagnosis (<50^a^ vs ≥ 50)	1.60, 0.71‐3.61 (0.31) n = 95	0.95, 0.41‐2.18 (0.99) n = 91
BMI (overweight/obese vs underweight/normal^a^)	0.64, 0.26‐1.61 (0.37) n = 95	0.94, 0.36‐2.41 (0.99) n = 91
Race (African American vs Caucasian^a^)	0.58, 0.22‐1.50 (0.33) n = 73	2.49, 0.90‐6.90 (0.09) n = 70
Ki67% (<50^a^ vs ≥ 50)	0.75, 0.24‐2.34 (0.77) n = 49	1.37, 0.40‐4.66 (0.76) n = 48
p53 (<10^a^ vs ≥ 10)	0.49, 0.19‐1.29 (0.23) n = 68	0.61, 0.23‐1.67 (0.45) n = 66
Neoadjuvant chemotherapy (platinum vs nonplatinum*)	4.01, 1.51‐10.68 (0.007**) n = 95	0.66, 0.27‐1.61 (0.37) n = 91

High change is change above the median (−0.75 for posttreatment‐baseline change in lymphocytes; −0.05 for posttreatment‐baseline change in monocytes). Odds are for change above the median.

^a^ Reference group.

** *P* < .05, n = sample size.

We further analyzed the relationship of changes in circulating lymphocytes and monocytes with outcomes according to the type of treatment patients received; platinum vs nonplatinum. There were no statistically significant associations between changes in monocytes or lymphocytes with pCR in either patients treated with platinum or nonplatinum regimens. However, patients treated with nonplatinum regimens who had a change in monocytes below the median (ie, greater decreases in monocytes) had improved DFS (*P* = .022, Figure S3A). There was no association between change in monocytes and DFS in patients treated with platinum (*P* = .88, Figure S3B). There were no associations with lymphocytes and DFS within platinum or nonplatinum groups (data not shown).

## 4 DISCUSSION

Our results suggest that transient lymphopenia from chemotherapy is not associated with clinical outcomes. However, we did find that lower absolute circulating monocyte counts after neoadjuvant chemotherapy were associated with better clinical outcomes. Though it is beyond the scope of our study to describe the underlying mechanism, prior studies have evaluated the role of the different subtypes of macrophages (circulating monocytes become macrophages when they enter tissues) in tumor progression. It is commonly described that M1 type macrophages have antitumor properties while the M2 type have tumor‐supporting properties. In a recent study, the relationship between OS and cluster of differentiation (CD) markers on tumor associated macrophages (TAMs) in the tumor stroma of breast cancer patients was evaluated. CD markers found on the M2 type, CD163, were associated with unfavorable OS, whereas CD markers on the M1 type, CD11c, were correlated with favorable OS in invasive breast cancer.^17^ Similarly, another study observed that higher numbers of M2 macrophages were strongly associated with proliferation, poor differentiation, estrogen receptor negativity, and histological ductal type (*P* < .001) and further demonstrated that breast cancer cells secreted factors that promote M2 differentiation.^18^ These results have also been observed in other types of cancers as well.^19^ Interestingly, it has also been observed that chemotherapy drives monocyte differentiation toward the M2 lineage.^20^ It is possible that the circulating monocytes we observed after neoadjuvant chemotherapy were differentiated into the M2 subtype, thus, explaining our findings that lower monocyte count was associated with better clinical outcomes. Indeed, other studies have demonstrated that higher numbers of TAMs are broadly correlated with hormone receptor negativity, adverse clinical, and pathological prognostic factors.^21^ The current findings are particularly interesting in the setting of a recent study which evaluated the TME in hormone receptor positive breast cancer before and after neoadjuvant chemotherapy.^22^ Tissue samples were assessed histologically, and whole transcriptome sequencing and panel RNA expression analysis were performed. Higher expression of M1 RNA was significantly correlated with more favorable response to neoadjuvant chemotherapy (*P* = .003), while lower M2 markers trended toward more favorable response to chemotherapy (*P* = .17). More research assessing the role of circulating immune cells using molecular assays is required to understand their role in tumor progression and the effects of neoadjuvant chemotherapy on clinical outcomes. Further, understanding the relationship of both the tumor microenvironment and surrogates of host immunity (as approximated in our study by circulating immune cells) may help in immune biomarker and immunotherapy development.

These findings can be interpreted in the context of other studies on circulating immune cells and cancer treatment. One study found that patients with TNBC with higher minimum absolute lymphocyte count had significantly lower overall mortality than those with lower minimum absolute lymphocyte count (HR = 0.23; 95% CI, 0.16‐0.35) and lower breast cancer mortality (HR = 0.19; CI, 0.11‐0.34).^23^ There were key differences in this study, perhaps most significantly that it included patients receiving both radiation therapy and adjuvant chemotherapy; radiation has been demonstrated to result in prolonged lymphopenia which may have more detrimental effects on clinical outcome. Indeed, another study evaluating the of effects of radiation of solid tumors on circulating lymphocytes showed that radiation‐induced lymphopenia was associated with persistent lymphopenia and increased risk of death.^14^ Similarly, another recent study showed that patients with solid tumors treated with PD‐1 inhibitors had differing lymphopenia frequency associated with previous treatments. Prior radiation was associated with a significantly higher frequency of lymphopenia, while prior chemotherapy was not, and the median time to progression was significantly shorter in patients with baseline lymphopenia.^13^ These data suggest that lymphopenia from different therapeutic modalities could have differing long‐term effects and clinical outcomes. In this context, our study suggests that lymphopenia due to chemotherapy might be more transient and shorter lasting than the lymphopenia induced by radiation. Further research is needed to determine if lymphopenia induced by different therapies have different prognoses in patients with cancer.

In our single‐site study, key advantages include a uniform breast cancer subtype, patient care and follow‐up; however, numerous limitations exist. The small sample size limits the power of some of our findings; albeit, the study population was focused on a specific breast cancer subtype. The data for this study was gathered retrospectively by a data warehouse and using chart review, thus, requiring additional validation studies. Furthermore, patients were treated with different neoadjuvant chemotherapies and there may have been variable effects on circulating lymphocytes and monocytes.

## 5 CONCLUSIONS

Our results suggest that monocytes may be correlated with response to chemotherapy and improved clinical outcomes. With further investigation into monocyte tissue differentiation, the role of circulating monocytes in disease response or progression could be elucidated and could act as a biomarker for predicting response to treatment and outcomes. Further, understanding key effector immune cells and their relationship to clinical outcomes may provide rationale for immunotherapy‐based treatment strategies (ie, targeting innate immune cell activation). Therefore, more research into effects of therapy on immune cell lines is essential to help further personalize treatment choices for individual patients.

## DATA SHARING

The data that support the findings of this study may be available from the corresponding author upon reasonable request; however, are subject to regulatory restrictions and approval.

## Conflict of Interest

CAS research funding Novartis, Tesaro, Pfizer, Medimmune; advisory board for Polyphor, Halozyme, Athenex, Bristol Meyers Squibb, and Genomic Health; RLBC research funding from Bristol Meyers; advisory board Bristol Meyers and Pfizer.

## Author Contributions

Sarah Talamantes: conceptualization, data curation, investigation, methodology, project administration, visualization, writing. Eric Xie: methodology, supervision, writing. Ricardo Costa: conceptualization, data curation, writing. Alfred Rademaker: formal analysis, visualization, writing. Melissa Chen: writing. Cesar Santa‐Maria: conceptualization, investigation, methodology, project administration, resources, supervision, visualization, writing.

## Supporting information

Fig S1A & B

Fig S1C & D

Fig S2A & B

Fig S2C & D

Fig S3A

Fig S3B
