# Supplementary figures and images for: Circulating immune cell dynamics in patients with triple negative breast cancer treated with neoadjuvant chemotherapy
Source: Cancer Med. 2020 Aug 5;9(19):6954–60. doi: 10.1002/cam4.3358 (PMC7541144; doi:10.1002/cam4.3358)

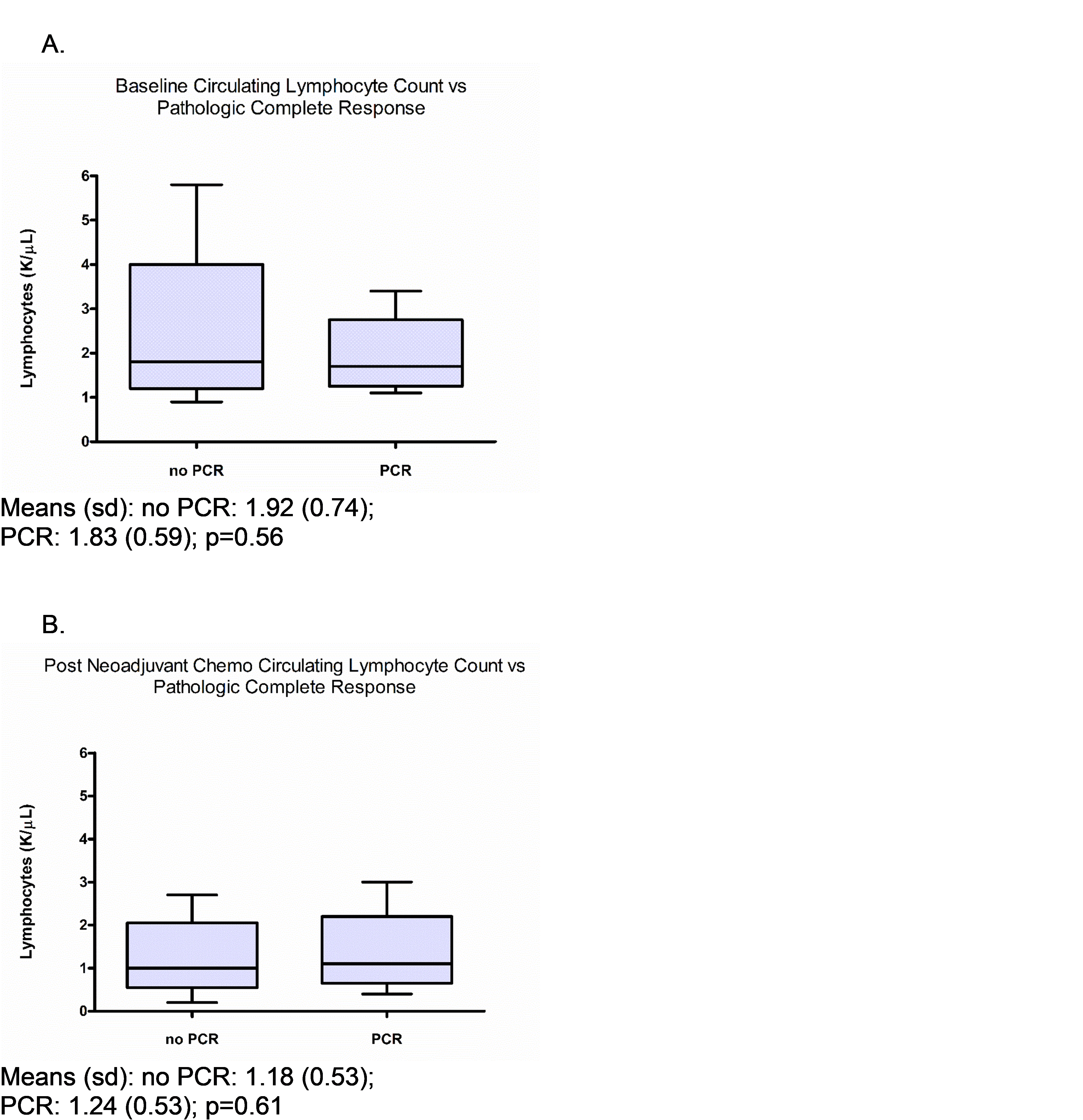

Supplement: Supplementary file 1 — Fig S1A & B [file CAM4-9-6954-s001.tif]

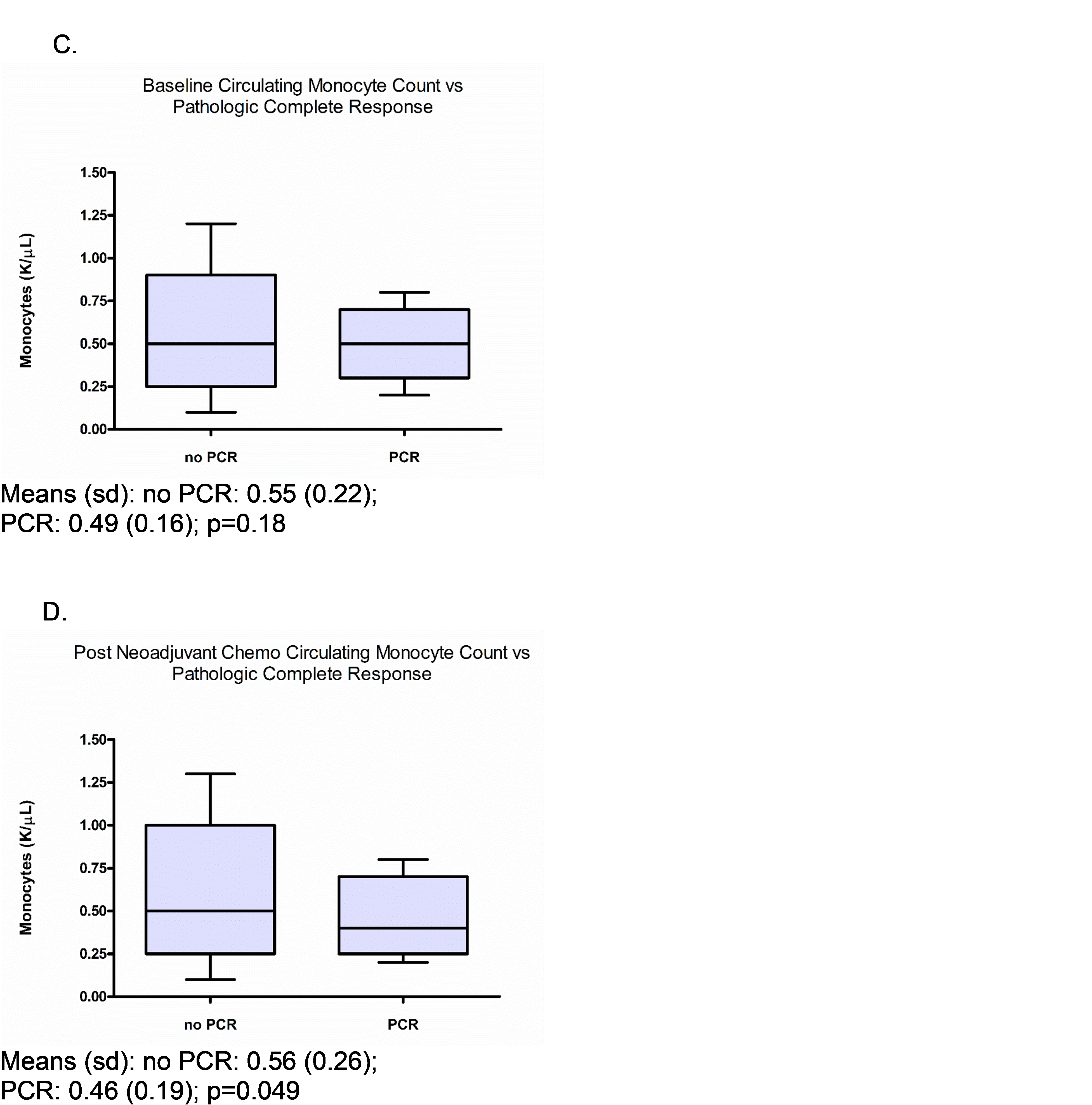

Supplement: Supplementary file 2 — Fig S1C & D [file CAM4-9-6954-s002.tif]

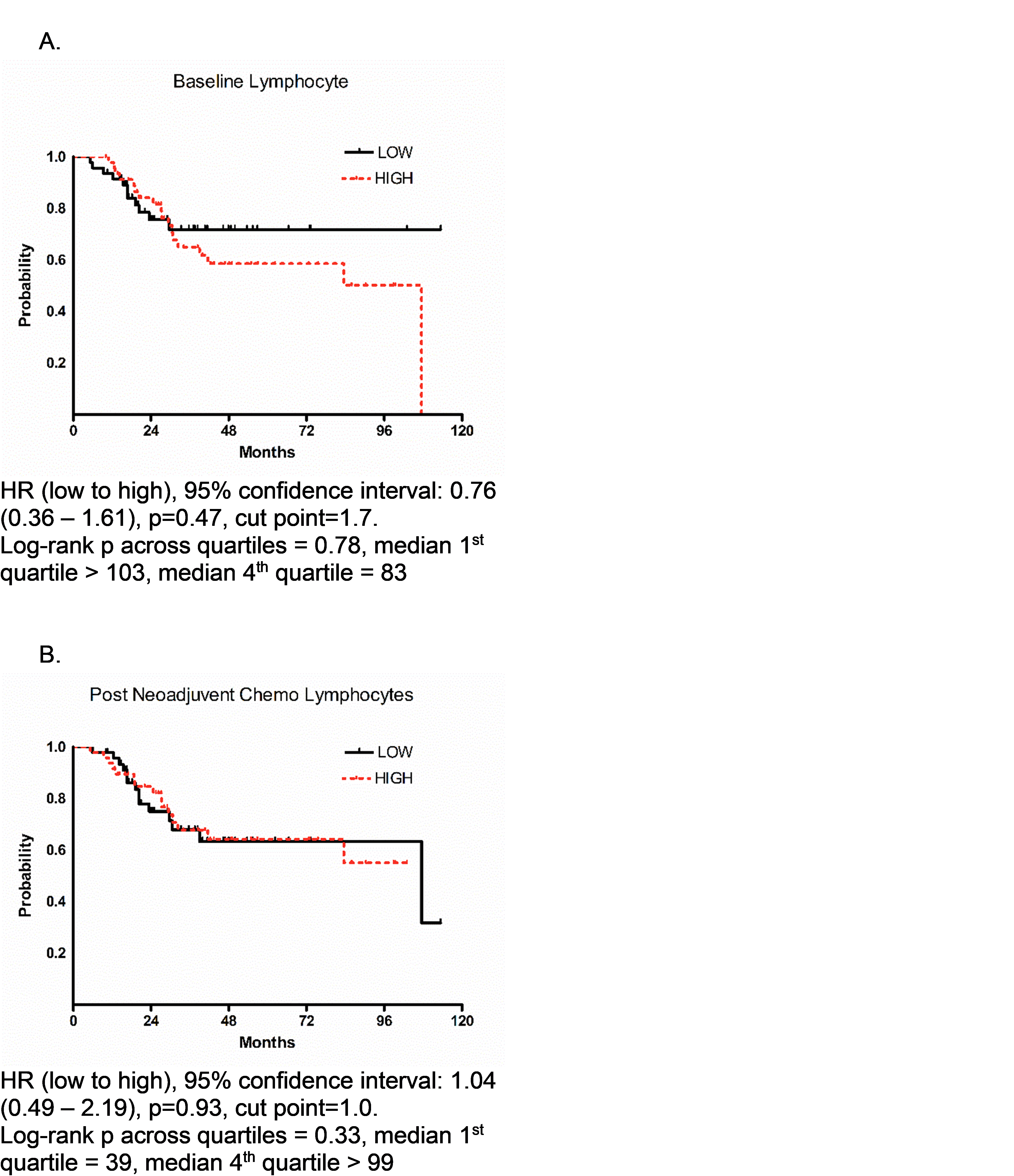

Supplement: Supplementary file 3 — Fig S2A & B [file CAM4-9-6954-s003.tif]

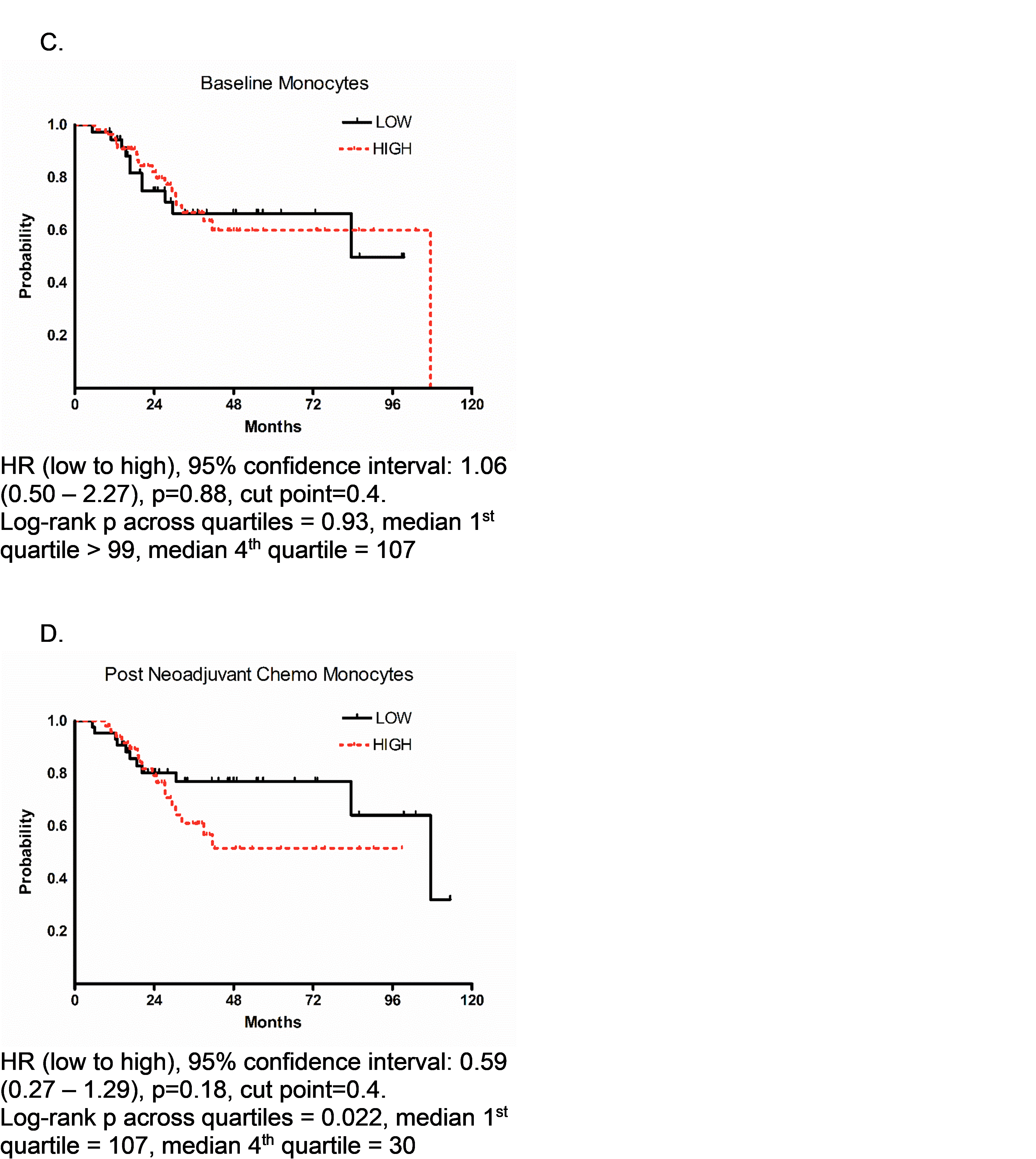

Supplement: Supplementary file 4 — Fig S2C & D [file CAM4-9-6954-s004.tif]

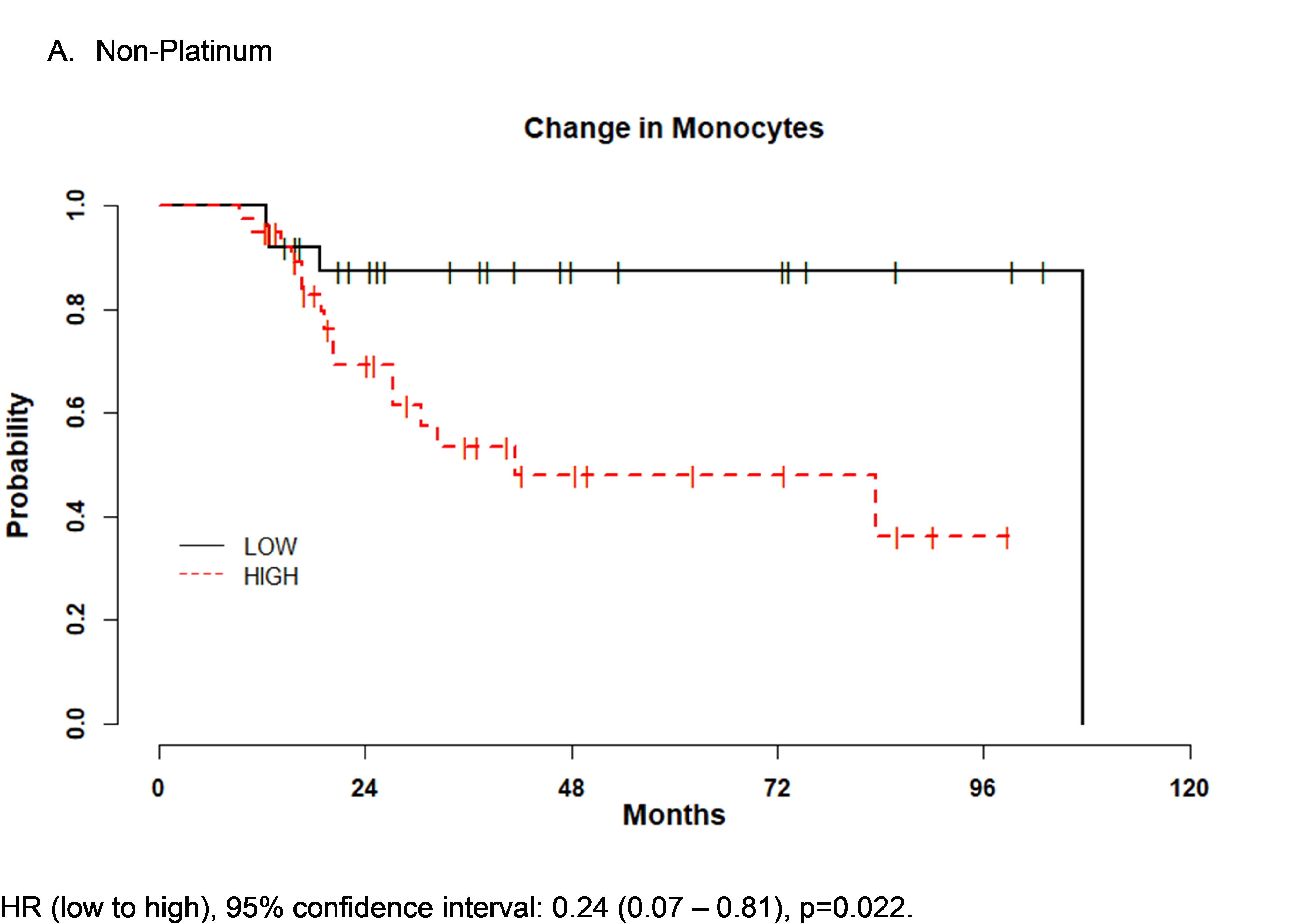

Supplement: Supplementary file 5 — Fig S3A [file CAM4-9-6954-s005.tif]

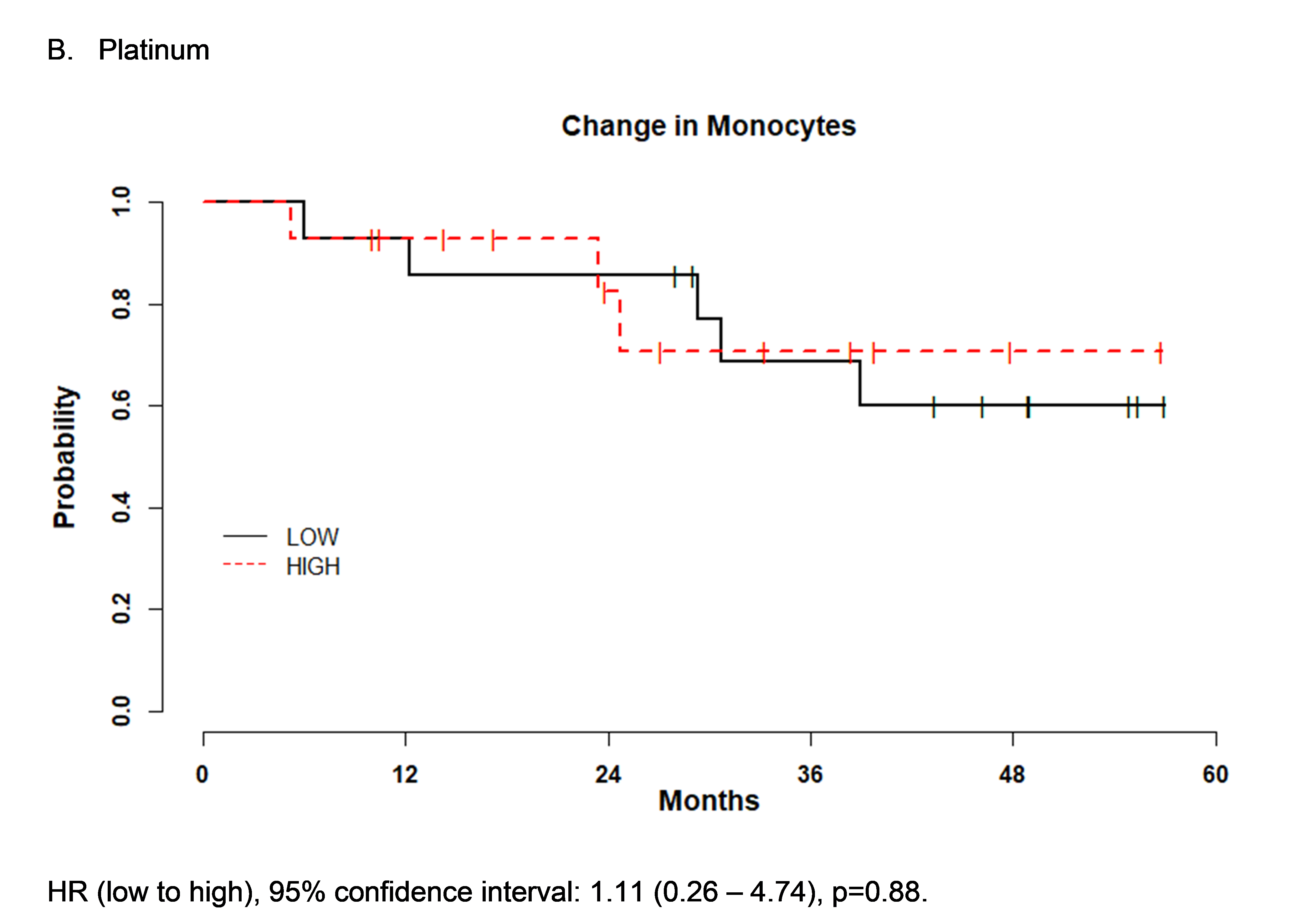

Supplement: Supplementary file 6 — Fig S3B [file CAM4-9-6954-s006.tif]
